# Compliance with Medication amongst Persons with Diabetes Mellitus during the COVID-19 Pandemic, Kerala, India: A Cross Sectional Study

**DOI:** 10.3390/tropicalmed7060104

**Published:** 2022-06-14

**Authors:** Ajan Maheswaran Jaya, Anthony D. Harries, Anisur Rahman, Mohammed Khogali, Palanivel Chinnakali, Bipin Gopal

**Affiliations:** 1 Directorate of Health Services, Kerala, Thiruvananthapuram 695101, India; bipingopal@yahoo.com; 2 Centre for Operational Research, International Union Against Tuberculosis and Lung Disease (The Union), 75001 Paris, France; adharries@theunion.org; 3 Department of Clinical Research, Faculty of Infectious and Tropical Diseases, London School of Hygiene and Tropical Medicine, London WC1E7HT, UK; 4 World Health Organization (WHO), Country Office, New Delhi 110029, India; rahmanan@who.int; 5 Special Programme for Research and Training in Tropical Diseases (TDR), World Health Organization, 1211 Geneva, Switzerland; khogalim@who.int; 6 Department of Preventive and Social Medicine, Jawaharlal Institute of Postgraduate Medical Education and Research, Puducherry 605006, India; palaniccm@gmail.com

**Keywords:** diabetes mellitus, COVID-19, India, Kerala, poor compliance with diabetes medication, primary health care facilities, SORT IT, operational research

## Abstract

Compliance with medication in persons with diabetes mellitus (DM) has been a challenge during the COVID-19 pandemic, leading to poor glycemic control and higher risk of complications. In the state of Kerala, India, 20–25% of adults have DM. Our cross-sectional study aimed to assess medication compliance and factors associated with poor compliance in DM persons attending selected primary care government facilities in Kerala during the COVID-19 pandemic. Persons registered with DM for >6 months were consecutively interviewed between August and September 2021. Poor compliance was defined as answering “No” to one or more of three questions related to access and intake of medication two weeks prior to and the day before the interview. Factors independently associated with poor compliance were assessed using adjusted prevalence ratios (aPr) and 95% confidence intervals. Of the 560 DM persons included, 209 (37%) exhibited poor compliance. Factors associated with poor compliance were age 19–45 years (aPr 1.4, 1.1–1.9); inability to be blood glucose tested during the COVID-19 pandemic (aPr 3.6, 2.9–4.3); not having COVID-19 (aPr 1.4, 1.0–1.9); and being double vaccinated against COVID-19 (aPr 1.4, 1.1–2.0). Focused attention must be paid to these groups to improve medication compliance and prevent DM complications and severe COVID-19-related disease.

## 1. Introduction

The coronavirus disease 2019 (COVID-19) pandemic continues unabated. As of the middle of March 2022, there have been 455 million confirmed cases and over 6 million deaths [1]. The pandemic has negatively impacted access to health care services, especially health services for people with chronic diseases all over the world [2]. National lockdowns, social restrictions, loss of livelihoods, patients’ concerns and fears about health facilities as places to contract COVID-19, overwhelmed health care providers dealing with COVID-19 cases and stretched health care systems have all led to severe disruptions in health services for other non-COVID-19 diseases, including the care and treatment for chronic disease.

One of the leading chronic non-communicable diseases is diabetes mellitus (DM). According to the International Diabetes Federation (IDF) Diabetes Atlas 2021 [3], an estimated 537 million adults are currently living with DM, representing 10% of the world’s population in the age group of 20–79 years. This number is predicted to rise to 643 million by 2030, and 783 million by 2045 [3]. Blood glucose levels in persons with DM can be effectively controlled by healthy lifestyle habits and compliance with or adherence to medication, using either oral hypoglycemic drugs and/or insulin and other injectable hypoglycemic agents. Adherence to safe and effective medications and a healthy lifestyle can reduce morbidity and mortality by preventing or delaying complications [4]. In a study in Saudi Arabia, the restrictions imposed by the COVID-19 pandemic negatively impacted DM care and significantly reduced compliance with medication and healthy lifestyle habits [5]. Declining medication compliance and less self-monitoring of blood glucose levels during the COVID-19 pandemic have also led to worse glycemic control among persons with DM [6].

India is one of the countries that is most severely affected by COVID-19, with over 43 million cases and 0.52 million COVID-19 related deaths [7]. Kerala, in southern India, was the first state to report COVID-19 cases, and to date, over 6.5 million cases have been notified [7]. The number of people with DM in India is 74.2 million, the second-highest in the world after China [3]. Within India, Kerala is one of the most advanced states in epidemiological transition [8]. An estimated 20–25% of the adult population are living with DM and require life-long medication and healthy life style guidance [8].

A study on medication adherence, using an 8-point Morisky scale, in persons with DM in rural Kerala in the pre-COVID-19 era demonstrated that 74% of the study population had poor adherence to medication, with contributory factors being poverty, use of oral hypoglycemic drugs, irregular blood glucose monitoring and limited education provided by health care professionals [9]. What has happened to persons with DM in primary care government facilities since the onset of the COVID-19 pandemic in Kerala is unknown. A recent study in Ethiopia during the COVID-19 pandemic demonstrated that nearly three-quarters of persons with DM who were assessed were poorly adherent to medication, with inability to attend health centers, co-morbidities and substance abuse being contributory factors [10]. The same situation may possibly exist in Kerala.

Although the terms adherence and compliance are often used interchangeably, they are different. Adherence reflects an active choice made by patients to agree to follow through with prescribed treatment or an agreed set of recommendations from a healthcare provider, while compliance is a passive behavior in which a patient follows a list of instructions from a health care worker about keeping appointments for follow-up or taking treatment as prescribed [11,12]. Given the complexities associated with measuring medication adherence [13], our study aimed to assess medication compliance and factors associated with poor compliance in persons with DM attending selected primary care government facilities in Kerala state, India, during the COVID-19 pandemic. Among persons with DM aged >18 years who attended these facilities between August and September 2021, the specific objectives were to document and assess: (i) baseline characteristics, awareness and knowledge of diabetes and patient status regarding COVID-19 vaccination and COVID-19 illness; (ii) the proportion of persons showing poor compliance to DM medication; and (iii) patient-specific factors associated with poor medication compliance.

## 2. Materials and Methods

### 2.1. Study Design

This was a cross-sectional study using data already collected as a result of interviews using a semi structured questionnaire, which were conducted during August and September 2021 during the COVID-19 pandemic.

### 2.2. General Setting

Kerala is a state in the southern part of India, with a literacy rate of 94% and a high human development index. The state has a total area of 38,863 sq. km and 33.4 million population, as per the national census 2011 [14]. The per capita Gross State Domestic Product (GSDP) of Kerala was estimated at about USD $3300 in 2019–20 [14]. There are 14 districts in Kerala. Thiruvananthapuram, which is the southernmost district and the capital of Kerala, where the study took place, is divided geographically into 19 health blocks, one urban and 18 rural.

The National Program for Prevention and Control of Cancer, Diabetes, Cardiovascular disease and Stroke (NPCDCS, 2010) started in Kerala in 2011. Since then, structured screening, diagnosis, treatment, and follow-up of DM are implemented at all government health facilities, including those at the primary care level. The Department of Health and Family Welfare, Kerala, has provided standardised treatment protocols for management of DM at primary care facilities. These centres serve as the first contact points for all beneficiaries and provide preventive, promotive and therapeutic services. According to treatment protocols, all drugs for treating DM (metformin, glimepiride, pioglitazone, and insulin) should be made available at these facilities free of cost. While patients are attending the primary care facilities, they can have blood glucose investigations performed at reduced rates fixed by the hospital management committees.

During the COVID-19 pandemic, the primary care facilities worked to the same schedules as in the pre-COVID era. However, during the peak of the second COVID-19 surge in Kerala, April to June 2021, primary health care facilities gave out repeat medications without the need to see a doctor. Furthermore, rapid response teams, which included primary care facility staff, took medications to the homes of those patients who were under quarantine and isolation.

### 2.3. Site Specific Settings, Sample Size, and Patient Interviews

From the 19 health blocks in the Thiruvananthapuram district, one urban and one rural health block were selected for the study. Poonthura is the only urban block, and it was therefore included. One rural block was selected by simple random sampling from the other 18 rural health blocks. All primary health care facilities in the two selected health blocks of Thiruvananthapuram district were included. The primary health care facilities comprise Primary Health centres (PHC), Community Health centres (CHC), and Family Health centres (FHC).

Based on sample size calculations used in the previous study in Kerala on drug adherence [9], it was estimated that 555 persons aged >18 years with DM would be required to study medication compliance. This sample was divided into 280 patients from each block. In Poonthura, which is the only urban block, there are four primary care facilities and thus 70 patients were included per facility. The selected rural block has seven primary health facilities and thus 40 patients were included per facility. Thus, in total, there were 11 primary health facilities selected for the study (3 PHCs, 2 CHCs and 6 FHCs).

Resources and health care workers available for DM diagnosis and care were similar in all the primary health facilities included in the study, except that CHCs had more medical officers and FHCs had extended working hours and the ability to measure glycosylated haemoglobin. From the selected facilities in the two health blocks, patients who were registered with DM for more than 6 months and visited the NCD clinics were consecutively interviewed between August and September 2021. After obtaining informed consent, the interviews were conducted as the patients exited their non-communicable disease (NCD) clinic after their routine visit.

The semi-structured interviews were conducted at the primary care facilities by trained data collectors under the supervision of the principal investigator (Ajan Maheswaran Jaya). A structured questionnaire was used after this had been pre-tested. Information was collected on demographic characteristics, socio-economic factors, DM and treatment related factors, co-morbidities, and family support for DM care. Patients were asked questions about their general knowledge and awareness of DM. Patients were also asked about medication collection and blood test follow up during the COVID-19 period as well as their status with COVID-19 vaccinations and COVID-19 infections.

Compliance to medication was measured by Yes/No answers to three questions about: (i) access to DM medicines during the COVID-19 pandemic; (ii) ability to take medicines every day during the last two weeks; and (iii) whether the DM medications were taken the day before the interview. Good compliance was defined as answering “Yes” to all three questions. Poor compliance was defined as answering “No” to one or more of the three questions. These three questions on compliance were chosen from a standard list [13] and were based on ease and rapidity with which to conduct exit interviews in persons with DM during the COVID-19 era.

Other information was also obtained on blood glucose monitoring, whether patients received any information or guidance on DM management and other relevant services from health care professionals. The data from the exit interviews were recorded in structured paper-based questionnaires.

### 2.4. Study Population and Study Period

The study population included registered persons aged > 18 years with DM who were undergoing treatment in 11 selected government health facilities in the Thiruvananthapuram district and who were interviewed between August and September 2021. For inclusion in the study, persons with DM had to have been on treatment at the primary health care facilities for at least 6 months.

### 2.5. Study Variables

Data variables for objective 1 included the socio-demographic and clinical characteristics of the persons registered with DM, as shown in Table 1. While most of these variables are self-explanatory, the occupation was defined as unemployed, manual labour (physical work conducted for daily wages) and other, which included skilled workers, clerks, professionals, and so on. Being below the poverty line referred to daily earnings being below a certain threshold, as defined by the Government of India. A smoking history was defined as current smoker (smoking one or more cigarettes a day), past smoker (smoked cigarettes in the past but not currently) and non-smoker (never smoked). The outcome variable for objective 2 and 3 included numbers with good compliance and poor compliance with medication, as defined earlier. The total number of registered DM patients under treatment in each facility was obtained from the DM register. All other data for the study were obtained from the patient questionnaire forms.

### 2.6. Analysis

Data from the paper-based questionnaires were entered in MS excel and were imported to Stata v13 (Stata Corporation College Station, College Station, TX, USA) for further cleaning and analysis. Descriptive statistics were used to describe the demographic and clinical characteristics of the patients and their medication compliance levels. Poor compliance with medication (defined as answering “No” to any one of the three questions) was the main outcome variable. To assess factors associated with poor compliance, the chi square test was used and unadjusted prevalence ratios with 95% confidence intervals (95% CI) were calculated. The multivariable analysis (Modified Poisson regression) was carried out, and adjusted prevalence ratios with 95% CI were calculated. Variables showing *p* < 0.2 in the unadjusted analysis were included in the multivariable model (to keep data content to an acceptable level, the *p*-values for the unadjusted analysis are not presented in the tables). The effects of any potential clustering at the primary health care level were accounted for during analysis. For all comparisons, levels of significance were set at <5% (*p* < 0.05).

## 3. Results

### 3.1. Socio-Demographic, Clinical Characteristics, and Health Care Processes

There were 560 persons with DM, whose sociodemographic and clinical characteristics are shown in Table 1. In brief, most patients were aged 46–69 years and there were more females than males. Most patients had attended school and were unemployed at the time of the study. Two thirds were below the poverty line and almost all were married. Two thirds of persons had a comorbidity, with the two commonest diseases being hypertension and dyslipidaemia. Most were non-smokers and did not drink alcohol.

Characteristics and management of DM are shown in Table 2. One third of persons had a family history of the disease. The median (inter quartile range) length of time since the diagnosis of diabetes had been made was 7 (3–12) years. Over half of the patients had been diagnosed and treated for over 6 years, and about two thirds were managed on single or multiple oral medications, with the majority purchasing their medications from government health facilities. Most persons had been told about how to take medication and the importance of regular doses, but despite this over two thirds had poor glycaemic control. Frequency of blood glucose testing and visits to primary care facilities were usually once a month.

Awareness levels among the study population about DM and its management is shown in Table 3. The positive responses to knowledge about DM being a chronic disorder, glycaemic control being important, and the symptoms and management of hypoglycaemia varied from 69% to 91%. However, less than half of the persons knew about target blood glucose levels or about how to control blood glucose levels.

COVID-19 related issues are shown in Table 4. A substantial proportion of persons with DM (80%) were able to have blood glucose levels tested during COVID-19 lockdowns. A small proportion (12%) had COVID-19. The majority (90%) had been vaccinated, with most of those receiving two doses of the vaccine.

### 3.2. Access and Compliance with DM Medication

Altogether, 351 (63%) persons with DM were compliant with DM medication. There were 209 (37%; 95% CI 33–42) persons who were non-compliant. Of these, there were four (<1%) who answered No to all three questions, 42 (7.5%) who answered No to two questions and 163 (29%) who answered No to one question. Among the persons with DM who answered No to one question, 72 (44%) answered No to access to DM medicines during the COVID-19 pandemic; 50 (31%) answered No to the ability to take medicines every day during the last two weeks; and 41 (25%) answered No to taking DM medications the day before the interview.

### 3.3. Factors Associated with Poor Compliance with DM Medication

All factors (with unadjusted and/or adjusted prevalence ratios) associated with poor compliance with medication (as defined under Section 3.2) are shown in the Supplementary Table S1. Table 5 provides the crude (unadjusted) and adjusted prevalence ratios for the variables showing *p* < 0.2 in the unadjusted analysis.

Based on adjusted prevalence ratios, being in the age group 19–45 years, not being able to be blood glucose tested during the COVID-19 era, and not having COVID-19, were associated with poor compliance with medication. Although on an unadjusted analysis, there was no effect of COVID-19 vaccination on compliance, after adjusting for possible confounders, having one or two doses of COVID-19 vaccine was associated with an increased prevalence of poor medication compliance.

## 4. Discussion

The key findings of this study were that just over one third of persons with DM were found to have poor compliance with medication during the COVID-19 era, with younger age, inability to be blood glucose tested, not having COVID-19 and being vaccinated against COVID-19 being independent risk factors for this association.

The finding that one third of our study population in Kerala showed poor compliance with medication during COVID-19 contrasts with the previous finding in Kerala in 2010 of a 74% poor adherence to medication [9]. At that time, it was likely that recommendations to improve medication adherence were put in place, and a further study in the same area in 2018 demonstrated considerable improvement with poor adherence being reduced to 40% [15]. Although compliance and adherence are different measurements, it was nevertheless encouraging to observe in our study that poor medication compliance was no higher than 40% during the COVID-19 period. It was also encouraging to observe that over 80% of persons with DM were able to have their blood glucose monitored and able to take their medications regularly during this difficult time, and these findings compare favourably with what was observed in Saudi Arabia during their COVID-19 lockdown [5]. It is possible that the practice of administering repeat medications without the need to see a doctor and the use of rapid response teams who took medicines to the homes of those with COVID-19 played a part in these results.

Why a younger age should be associated with poor medication compliance is unclear, although this finding has been reported elsewhere and may be due to work-related or family-related issues taking precedence over health care needs [16,17].

It is intuitive that the inability to have blood glucose testing performed during the COVID-19 pandemic should be associated with poor compliance as blood glucose monitoring and regular taking of medication are associated. This association has also been reported elsewhere [6].

The observation that the small number of those who contracted COVID-19 had better compliance has not been made previously. There are several possible reasons for this association. People who are willing to go out to the community for shopping, exercise and health care activities, such as visiting health facilities to collect medication and receive care, may be the same people who become more exposed to SARS-CoV-2 and therefore are infected. Good compliance with medication might also be associated with contracting COVID-19. There is an increased risk of SARS-CoV-2 in hospital and health care settings, particularly for those who are not well protected against viral airborne transmission [18,19]. There is good evidence that outpatient clinic rooms have poor ventilation systems [20], and an assessment of DM clinics in China demonstrated inadequate implementation of measures to prevent airborne transmission of respiratory pathogens [21].

Being vaccinated against COVID-19 with one or two doses appeared to be associated with poor medication compliance. The reasons for this are unclear. However, this is a statistical association. Operationally, and in the middle of a pandemic, we would not be encouraging at risk persons such as those with DM not to become vaccinated. There are data to suggest that persons with DM are at increased risk of COVID-19 [22], and there is strong evidence that those with DM are at much higher risk of severe disease requiring hospitalisation, multi-organ failure, coagulopathy and death compared with those who do not have DM [17,23].

The strengths of this study were the good sampling technique, the large numbers of persons with DM interviewed, few missing data and the conduct and reporting of the study according to the STROBE guidelines (Strengthening the Reporting of Observational Studies in Epidemiology) statement [24].

There were, however, some limitations. First, we decided to measure compliance rather than adherence to medication, as our methodology was more directed towards assessing compliance. However, the tool we used to measure compliance had not been previously validated and was chosen from a standard list of questions. This was to make it quick and easy to interview persons with DM about compliance in the COVID-19 era where physical and social distancing was important to maintain. Second, this was a cross-sectional survey meaning that it is not possible to assess causal relationships, and some of the associations found in this study (such as poor compliance associated with COVID-19 vaccination) must be viewed with caution. Third, the study was limited by its focus on medication and blood glucose control. Many of the patients had at least one additional comorbidity besides DM. The effect of these comorbidities and their treatment approaches (lifestyle optimization, weight loss, antihypertensive drugs and statins) were not addressed in our study, yet could have added a more comprehensive and multifactorial perspective. Finally, the study was also pragmatic and based on exit-interviews, so there were a number of variables of interest such as anthropometric and laboratory measurements that we did not record, and yet these might have been useful for a deeper understanding of the issues involved.

Despite the limitations, there are a number of important implications from this study. First, it is clear from the demographic and clinical characteristics of the study population that more needs to be achieved about education of DM and improving knowledge about self-management. Despite the fact that most people had been to school and had DM for over 6 years, more than half did not know about target blood glucose levels or about how to better control these levels and, as a result, overall glycaemic control was poor. Better knowledge, better adherence and compliance with medication and better DM control can prevent and reduce serious complications from this disease [4]. Furthermore, DM persons with well-controlled blood glucose levels have significantly lower mortality rates from COVID-19 compared with those whose levels are poorly controlled [23]. Attention to associated co-morbidities such as hypertension will also help to reduce cardiovascular complications.

Second, it is particularly important to target the younger age group aged 19–45 years, and despite their pressures of family-life and work, impress on them the importance of better medication compliance. This can be helped by considering easier home-based care models using more self-monitoring and possible use of telemedicine for those who might have access to wireless or mobile phone networks. Such an approach in turn would keep people with DM away from busy crowded health facilities where they are at risk of catching not only COVID-19, but other dangerous respiratory infections such as tuberculosis [25].

Third, for those persons with DM who need to regularly visit health facilities for health care checks, blood glucose measurements and medication, it is imperative that the health sector makes these visits as safe as possible and pays due attention to good quality infection prevention control procedures. Although only 12% of the study cohort contracted COVID-19, this is a group at high risk of severe illness and death [17,23]. Serious efforts must be made to ensure the outpatient infrastructure and ventilation systems are as good as possible and that face masks, physical distancing and eye protection are used, as these have been demonstrated to significantly reduce the risk of COVID-19 transmission [26]. It is also important that the health sector persuades the small minority of DM persons who were unvaccinated in our study to take up the vaccination. Other measures to make health visit attendance safer are to require those with stable DM to attend less frequently and support more peripheral centres to become drug dispensing units and thereby prevent overcrowding.

## 5. Conclusions

In conclusion, 560 persons with DM were interviewed during the COVID-19 pandemic between August and September 2021. Of these, about one third were assessed as being non-compliant with medication. Key risk factors for non-compliance included being of a younger age and not having their blood glucose levels monitored during the COVID-19 era. More attention must be paid to these groups so that they can better comply with medication which in turn should prevent or reduce their risk of DM complications and more severe disease resulting from COVID-19. Two important take home messages are the need to educate and improve knowledge about the self-management of DM, especially amongst the younger generation, and to ensure that good infection, prevention and control activities are always implemented in the health facilities that are attended by persons with DM.

## Open Access Statement and Disclaimer

In accordance with WHO’s open-access publication policy for all work funded by WHO or authored/co-authored by WHO staff members, WHO retains the copyright of this publication through a Creative Commons Attribution IGO license (http://creativecommons.org/licenses/by/4.0/igo/legalcode) which permits unrestricted use, distribution and reproduction in any medium, provided the original work is properly cited. There should be no suggestion that WHO endorses any specific organization, products or services. The views expressed in this article are those of the authors and do not necessarily reflect those of their affiliated institutions. The use of the WHO logo is not permitted. This notice should be preserved along with the article’s original URL.

## Figures and Tables

**Table 1:** Socio-demographic and clinical characteristics in persons with diabetes mellitus attending primary health care facilities, Kerala, India, 2021.

Category	Variables	Number	(%)
Total		560	
Age group years	19–45	65	(12)
	46–69	404	(72)
	≥70	91	(16)
Gender	Male	228	(41)
	Female	332	(59)
Education	No formal schooling	42	(8)
	School	426	(76)
	University	92	(16)
Occupation	Unemployed	322	(58)
	Manual labourer	98	(18)
	Other	149	(25)
Socio-economic status	Below poverty line	351	(63)
	Above poverty line	209	(37)
Marital status	Married	540	(96)
	Single	20	(4)
Comorbidity *	Hypertension	264	(47)
	Coronary heart disease	58	(10)
	Stroke	8	(1)
	Bronchial asthma	20	(4)
	Dyslipidaemia	216	(39)
	Thyroid disease	59	(11)
	None	188	(34)
Smoking history	Current	13	(2)
	Past smoker	19	(4)
	Non-smoker	528	(94)
Alcohol	Use in the last 12 months	60	(11)
Smokeless tobacco	Use in the last one month	22	(4)

Footnote: *comorbidity = any other non-communicable disease in addition to diabetes mellitus, numbers add up to >560 because several patients had more than one comorbidity.

**Table 2:** Characteristics and management of DM in persons attending primary health care facilities, Kerala, India, 2021.

Category	Variables	Number	(%)
Total		560	
Family history of DM	Positive family history	186	(33)
Duration of DM treatment in years	<2	49	(9)
	2–5	202	(36)
	≥6	309	(55)
Type of DM treatment	Oral medication single	142	(25)
	Oral medication multiple	232	(41)
	Oral medication and insulin	139	(25)
	Insulin	47	(9)
Location of primary health facility	Urban	276	(49)
	Rural	284	(51)
Place of purchase of medicines	Government	507	(90)
	Private	9	(2)
	Government and Private	44	(8)
Did the health worker explain	How to take medicines (response = Yes)	521	(93)
	The need to take medicines regularly (response = Yes)	306	(55)
Glycemic control **^,†^	Good	169	(32)
	Poor	362	(68)
Frequency of blood glucose tests	Once a month	450	(80)
	Once in two months	53	(9)
	More than 2 months	58	(11)
Frequency of visit to PHC	Once a month	503	(90)
	Once in two months	23	(4)
	More than 2 months	34	(6)

Footnotes: DM = diabetes mellitus; PHC = primary health centre; ** good glycemic control = fasting blood glucose of 80–130 mg/dL or postprandial blood glucose < 180 mg/dL; poor glycemic control = fasting blood glucose of >130 mg/dL or postprandial blood glucose > 180 mg/dL; ^†^ 29 missing values.

**Table 3:** Awareness about diabetes mellitus in persons with diabetes mellitus attending primary health care facilities, Kerala, India, 2021.

Questions Asked to Persons with DM	Number Responding Yes	(%)
Total	560	
Is DM a chronic disorder?	489	(87)
Is glycemic control important?	509	(91)
Do you know about target blood glucose levels?	227	(41)
Do you know about symptoms of hypoglycaemia?	386	(69)
Do you know how to manage hypoglycaemia?	429	(77)
Do you know how to control blood glucose levels in DM?	175	(31)

Footnote: DM = diabetes mellitus.

**Table 4:** COVID-19 related issues in persons with diabetes mellitus attending primary health care facilities, Kerala, India, 2021.

Questions Asked to Persons with DM	Number Responding Yes	(%)
Total	560	
Could you get blood glucose tested during COVID-19 lockdown in 2021?	447	(80)
Did you have COVID-19 confirmed by LFA/RT-PCR?	68	(12)
Have you had COVID-19 vaccination—One dose only	168	(30)
Have you had COVID-19 vaccination—Two doses	337	(60)
At the time of the interview were you unvaccinated?	55	(10)

Footnote: DM = diabetes mellitus; LFA = lateral flow antigen test; RT-PCR = real time polymerase chain reaction.

**Table 5:** Factors associated with poor medication compliance in persons with diabetes mellitus attending primary health care facilities, Kerala, India, 2021.

Category	Variables	Total	Poor Medication Compliance		Crude Prevalence Ratio (95% CI)	Adjusted Prevalence Ratio (95% CI)	*p*-Value *
			*n*	(%)			
Total		560	209	(37)			
Age group years	19–45	65	30	(46)	1.3 (0.9, 1.8)	1.4 (1.1, 1.9)	0.02
	46–69	404	142	(35)	Ref		
	≥70	91	37	(41)	1.2 (0.9, 1.5)	0.9 (0.8, 1.3)	0.97
Occupation	Unemployed	322	134	(42)	1.6 (1.1, 2.2)	1.3 (0.9, 1.9)	0.26
	Manual labourers	98	26	(27)	Ref		
	Others	140	49	(35)	1.2 (0.9, 1.9)	1.2 (0.8, 1.6)	0.42
Socio-economic	Below poverty line	351	72	(21)	Ref		
	Above poverty line	209	137	(66)	3.2 (2.5, 4.0)	1.0 (0.9, 1.2)	0.68
Alcohol use	In last 12 months—Yes	60	17	(28)	Ref		
	In last 12 months—No	500	192	(38)	1.4 (0.9, 2.1)	0.9 (0.7, 1.4)	0.87
Type of DM treatment	Oral medication single	142	70	(49)	1.7 (1.0, 2.7)	1.5 (0.9, 2.1)	0.06
	Oral medication multiple	232	82	(35)	1.2 (0.7, 1.9)	1.3 (0.9, 1.8)	0.26
	Oral medication + insulin	139	43	(31)	1.0 (0.6, 1.7)	1.2 (0.8, 1.8)	0.44
	Insulin only	47	14	(30)	Ref		
Place of purchase of medicines	Government	507	178	(35)	Ref		
	Private	9	5	(56)	1.3 (0.7, 2.5)	1.3 (0.8, 2.1)	0.32
	Government/Private	44	26	(59)	1.7 (1.3, 2.2)	1.1 (0.9, 1.5)	0.23
Did health worker explain	How to take medicines—Yes	521	190	(37)	Ref		
	How to take medicines—No	39	19	(49)	1.3 (0.9, 1.9)	0.9 (0.7, 1.3)	0.63
Frequency of blood glucose tests	Once a month	450	159	(35)	1.0 (0.7, 1.5)	1.3 (0.8, 1.9)	0.27
	Once in 2 months	52	18	(35)	Ref		
	More than 2 months	58	32	(55)	1.6 (1.0, 2.5)	1.1 (0.7, 1.7)	0.56
Frequency of visits to PHC	Once a month	503	177	(35)	1.0 (0.6, 1.8)	0.8 (0.5, 1.4)	0.52
	Once in 2 months	23	8	(35)	Ref		
	More than 2 months	34	24	(71)	2.0 (1.1, 3.7)	0.9 (0.5, 1.7)	0.89
**Questions to Patients about DM**							
Blood glucose control	Important—Yes	509	183	(36)	Ref		
	Important—No	51	26	(51)	1.4 (1.1, 1.9)	1.1 (0.8, 1.5)	0.61
Can you control blood glucose levels	Yes	175	58	(33)	Ref		
	Partially	368	141	(38)	1.2 (0.9, 1.5)	0.9 (0.7, 1.1)	0.26
	No	17	10	(59)	1.8 (1.1, 2.8)	1.1 (0.7, 1.8)	0.66
**Questions to Patients about COVID-19**							
Was blood glucose tested	During the pandemic—Yes	447	109	(24)	Ref		
	During the pandemic—No	113	100	(89)	3.6 (3.0, 4.3)	3.6 (2.9, 4.3)	<0.001
Have you had COVID-19	Yes	68	20	(29)	Ref		
	No	492	189	(38)	1.3 (0.9, 1.9)	1.4 (1.0, 1.9)	0.03
Have you had COVID-19 vaccination	Did not receive vaccine	55	20	(36)	Ref		
	Received one dose	168	71	(42)	1.2 (0.8, 1.7)	1.5 (1.1, 2.1)	0.012
	Received two doses	337	118	(35)	0.9 (0.7, 1.4)	1.4 (1.1, 2.0)	0.024

Footnotes: DM = diabetes mellitus; HCW = health care workers; * *p*-value from multivariable regression analysis (modified Poisson model). Variables which had *p*-value < 0.2 in the unadjusted analysis were included in the multivariable model.

## Data Availability

The data for this paper are available upon request from the principal investigator.

## Supplementary Materials

The supplementary material is available online at https://www.mdpi.com/article/10.3390/tropicalmed7060104/s1; Supplementary Table S1: Factors associated with poor medication compliance in persons with diabetes mellitus attending primary health care facilities, Kerala, India, 2021.

## Open Access Statement and Disclaimer

In accordance with WHO’s open-access publication policy for all work funded by WHO or authored/co-authored by WHO staff members, WHO retains the copyright of this publication through a Creative Commons Attribution IGO license (http://creativecommons.org/licenses/by/3.0/igo/legalcode) which permits unrestricted use, distribution and reproduction in any medium, provided the original work is properly cited. There should be no suggestion that WHO endorses any specific organization, products or services. The views expressed in this article are those of the authors and do not necessarily reflect those of their affiliated institutions. The use of the WHO logo is not permitted. This notice should be preserved along with the article’s original URL.
