# Supplementary material for: Compliance with Medication amongst Persons with Diabetes Mellitus during the COVID-19 Pandemic, Kerala, India: A Cross Sectional Study
Source: Trop Med Infect Dis. 2022 Jun 14;7(6):104. doi: 10.3390/tropicalmed7060104 (PMC9228986; doi:10.3390/tropicalmed7060104)
Supplement: Supplementary file 1 [file tropicalmed-07-00104-s001.zip › tropicalmed-1737686-SI.pdf]

Tropical Medicine and Infectious Disease

Supplementary Material: Factors Associated with Poor Medication Compliance in Persons with Diabetes Mellitus Attending Primary Health Care Facilities, Kerala, India, 2021

Ajan Maheswaran Jaya [1,*], Anthony D. Harries [2,3], Anisur Rahman [4], Mohammed Khogali [5], Palanivel Chinnakali [6] and Bipin Gopal [1]

Table S1. Factors associated with poor medication compliance in persons with diabetes mellitus attending primary health care facilities, Kerala, India, 2021.

Category	Variables	Total	Poor Medication Compliance		Crude Prevalence Ratio (95% CI)	Adjusted Prevalence Ratio (95% CI)	*p* Value
			n	(%)			
	**Total**	**560**	**209**	**(37)**			
Age group years	19–45	65	30	(46)	1.31 (0.98, 1.76)	**1.43 (1.07, 1.93)**	**0.02**
	46–69	404	142	(35)	Ref		
	≥ 70	91	37	(41)	1.15 (0.87, 1.53)	0.99 (0.77, 1.28)	0.97
Gender	Male	228	81	(36)	Ref		
	Female	332	128	(39)	1.09 (0.87, 1.35)		
Education	No formal schooling	42	18	(43)	1.17 (0.81, 1.70)		
	School	426	156	(37)	Ref		
	University	92	35	(38)	1.04 (0.78, 1.39)		
Occupation	Unemployed	322	134	(42)	1.57 (1.10, 2.24)	1.32 (.95, 1.86)	0.26
	Manual labourers	98	26	(27)	Ref		
	Others	140	49	(35)	1.15 (0.89, 1.97)	1.16 (0.81, 1.65)	0.42
Socio-economic	Below poverty line	351	72	(21)	Ref		
	Above poverty line	209	137	(66)	3.20 (2.54, 4.02)	1.04 (0.85, 1.18)	0.68
Marital status	Married	540	200	(37)	Ref		
	Single	20	9	(45)	1.21 (0.73, 1.99)		
Comorbidities	Hypertension—Yes	264	103	(39)	1.09 (0.88, 1.35)		
	Hypertension—No	296	106	(36)	Ref		
	Coronary heart disease—Yes	58	22	(38)	1.01 (72, 1.44)		
	Coronary heart disease—No	502	187	(37)	Ref		
	Dyslipidaemia—Yes	216	77	(36)	Ref		
	Dyslipidaemia—No	344	132	(38)	1.08 (0.86, 1.35)		
	Thyroid disease—Yes	59	26	(44)	1.21 (0.89, 1.65)		
	Thyroids disease—No	501	183	(37)	Ref		
Number of comorbidities	None	188	69	(37)	1.09 (0.86, 1.38)		
	1 to 2	350	118	(34)	Ref		
	> 2	55	22	(40)	1.19 (0.83, 1.69)		
Smoking status	Non-smoker	528	198	(38)	1.19 (0.61, 2.32)		
	Current	13	5	(39)	1.22 (0.47, 3.16)		
	Past smoker	19	6	(32)	Ref		
Alcohol use	In last 12 months—Yes	60	17	(28)	Ref		
	In last 12 months—No	500	192	(38)	1.35 (0.89, 2.06)	0.97 (0.66, 1.43)	0.87
Smokeless tobacco	In last 1 month—Yes	22	9	(41)	1.1 (0.66, 1.84)		
	In last 1 month—No	538	200	(37)	Ref		
Family History of DM	Yes	186	67	(36)	1.05 (0.84, 1.33)		
	No	374	142	(38)	Ref		
Location of health facility	Urban	276	97	(35)	Ref		

	Rural	284	112	(39)	1.12 (0.91, 1.39)		
Duration of DM since diagnosis in years	< 2	41	17	(42)	1.12 (0.76, 1.66)		
	2–5	194	72	(37)	1.01 (0.80, 1.27)		
	≥ 6	325	120	(37)	Ref		
Duration of DM treatment in years	< 2	49	21	(43)	1.13 (0.79, 1.64)		
	2–5	202	76	(38)	Ref		
	≥ 6	309	120	(39)	1.1 (0.78, 1.57)		
Type of DM treatment	Oral medication single	142	70	(49)	1.65 (1.04, 2.65)	1.45 (0.98, 2.14)	0.06
	Oral medication multiple	232	82	(35)	1.18 (0.74, 1.90)	1.25 (0.85, 1.84)	0.26
	Oral medication + insulin	139	43	(31)	1.04 (0.62, 1.72)	1.17 (0.78, 1.76)	0.44
	Insulin only	47	14	(30)	Ref		
Place of purchase of medicines	Government	507	178	(35)	Ref		
	Private	9	5	(56)	1.29 (0.67, 2.49)	1.33 (0.84, 2.14)	0.32
	Government/Private	44	26	(59)	1.68 (1.28, 2.20)	1.13 (0.88, 1.47)	0.23
Did health worker explain	How to take medicines—Yes	521	190	(37)	Ref		
	How to take medicines—No	39	19	(49)	1.34 (0.95, 1.88)	0.92 (0.66, 1.28)	0.63
Did health worker explain	Regular medicine—Yes	306	108	(35)	Ref		
	Regular medicine—No	254	101	(40)	1.12 (0.90, 1.34)		
Glycemic control ǂ (n = 531)	Good	169	68	(40)	1.13 (0.90, 1.43)		
	Poor	362	128	(35)	Ref		
Frequency of blood glucose tests	Once a month	450	159	(35)	1.02 (0.69, 1.51)	1.25 (.84, 1.85)	0.27
	Once in 2 months	52	18	(35)	Ref		
	More than 2 months	58	32	(55)	1.59 (1.03, 2.47)	1.14 (0.74, 1.73)	0.56
Frequency of visits to PHC	Once a month	503	177	(35)	1.01 (0.57, 1.79)	0.84 (0.50, 1.42)	0.52
	Once in 2 months	23	8	(35)	Ref		
	More than 2 months	34	24	(71)	2.03 (1.11, 3.70)	0.96 (0.54, 1.69)	0.89
**Questions to Patients about DM**							
DM a chronic disorder	Yes	489	180	(37)	Ref		
	No	71	29	(41)	1.11 (0.82, 1.5)		
Blood glucose control	Important—Yes	509	183	(36)	Ref		
	Important—No	51	26	(51)	1.42 (1.06, 1.90)	1.08 (0.80, 1.48)	0.61
Blood glucose level targets	Do you know them—Yes	227	86	(38)	1.03 (0.82, 1.28)		
	Do you know them—No	333	123	(37)	Ref		
Hypoglycemia symptoms	Do you know them—Yes	386	141	(37)	Ref		
	Do you know them—No	174	68	(39)	1.07 (0.85, 1.34)		
Hypoglycemia management	Do you know—Yes	429	155	(36)	Ref		
	Do you know—No	131	54	(41)	1.14 (0.90, 1.45)		
Can you control blood glucose levels	Yes	175	58	(33)	Ref		
	Partially	368	141	(38)	1.15 (0.9, 1.5)	0.87 (0.69, 1.11)	0.26
	No	17	10	(59)	1.77 (1.13, 2.78)	1.11 (0.69, 1.78)	0.66
**Questions to Patients about COVID-19**							
Was blood glucose tested	During the pandemic—Yes	447	109	(24)	Ref		
	During the pandemic—No	113	100	(89)	3.63 (3.04, 4.33)	**3.55 (2.94, 4.31)**	**<0.001**
Have you had COVID-19	Yes	68	20	(29)	Ref		
	No	492	189	(38)	1.31 (0.89, 1.92)	**1.40 (1.02, 1.91)**	**0.03**
Have you had COVID-19 vaccination	Did not receive vaccine	55	20	(36)	Ref		
	One dose	168	71	(42)	1.16 (0.79, 1.72)	**1.51 (1.09, 2.08)**	**0.012**
	Two doses	337	118	(35)	0.96 (0.66, 1.44)	**1.44 (1.05, 1.98)**	**0.024**

Footnotes: DM = diabetes mellitus; HCW = health care workers; **p* value from multivariable regression analysis (modified Poisson model). Variables which had *p* value < 0.2 in the unadjusted analysis were included in the multivariable model. ǂ Good glycemic control: fasting blood glucose of 80–130 mg/dL or Postprandial blood glucose < 180 mg/dL; Poor glycemic control: fasting blood glucose of > 130 mg/dL or Postprandial blood glucose > 180 mg/dL; † 29 missing values .
